# Methods for estimating milk production of dairy cows with full cow-calf contact

**DOI:** 10.3168/jdsc.2026-1017

**Published:** 2026-04-23

**Authors:** C.L. van Zyl, A.T.M. van Knegsel, S. Agenäs, E.A.M. Bokkers

**Affiliations:** 1 Adaptation Physiology Group, Wageningen University & Research, 6700 AH Wageningen, the Netherlands; 2 Animal Production Systems Group, Wageningen University & Research, 6700 AH Wageningen, the Netherlands; 3 Department of Applied Animal Science and Welfare, Swedish University of Agricultural Sciences, 750 07 Uppsala, Sweden; 4 The Beijer Laboratory for Animal Science, Faculty for Veterinary Medicine and Animal Science, SLU, 750 07 Uppsala, Sweden

## Abstract

Dairy cows with full cow-calf contact can nurse their calves and perform maternal behaviors without hindrance. Owing to the unrestricted suckling, the total milk production of the suckled cows (i.e., machine milk yield [MMY] and milk consumed by the calf) is unknown, albeit required for performance testing and sustainability analyses of prolonged cow-calf contact. This study aimed to estimate the daily milk production of dairy cows with full cow-calf contact spanning the whole day using 2 methods. First, individual milk production was estimated using the daily energy intake during the preweaning suckling period and the ratio of MMY to the previous day's energy intake, calculated for the 8-wk postsuckling period. Second, individual milk production was estimated using the daily MMY during the suckling period and adding the estimated calf milk consumption based on their body weight, growth, and energy intake from concentrates. This was done for conventionally managed cows with no contact with their calves beyond 13.5 h after birth (NC; n = 20) and for cows with full contact with their calves until gradual weaning at 13.0 ± 1.1 wk of age (FC; n = 18). Using the first method based on energy intake of the cow, milk production of NC cows was estimated at 33.33 ± 1.40 kg/d and their milk yield to the automatic milking unit averaged 37.73 ± 1.40 kg/d during the first 12 wk of lactation. This discrepancy might be because cows likely experienced a negative energy balance during the preweaning period, which was not accounted for in the estimation. For FC cows, average milk production was estimated at 35.30 ± 1.40 kg/d and their milk yield to the milking unit averaged 24.74 ± 1.60 kg/d during the suckling period of the first 12 wk of lactation. Using the second method based on the estimated milk consumption of FC calves (14.91 ± 2.9 kg/d; mean ± SD), milk production of FC cows during the suckling period was estimated at 37.94 ± 1.80 kg/d. In conclusion, both methods evaluated could estimate milk production of suckled FC cows. In the first method, milk production was underestimated when using ME intake and milk yield during the nonsuckling period, possibly due to not accounting for body reserve mobilization. In the second method, milk production of suckled cows estimated using calf growth aligned with the recorded MMY of nonsuckled cows. The ∼15 kg/d estimated milk consumption of FC calves during the first 13 wk of life corresponded to the recorded milk yield difference between FC and NC cows. Methods evaluated in this study can be applied for performance testing and sustainability analyses of cow-calf contact systems.

## Highlights

• Total milk production of cows whose calves can suckle unrestrictedly is unknown.
• Milk production estimated from cow energy intake was underestimated.
• Milk production estimated from calf growth aligned with yield of nonsuckled cows.
• Calves were estimated to consume ~15 kg milk/day during the 13-week suckling period.
• Estimations did not consider calves' roughage intake and ingested milk composition.

## Graphical Abstract

**Figure:**
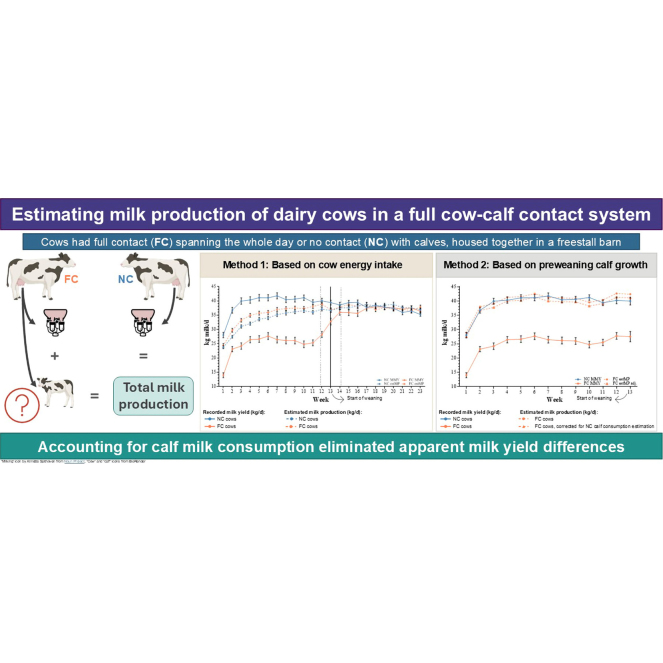
**Summary:**Total milk production of dairy cows with full calf contact is unknown, as calves can suckle throughout the whole day. We aimed to estimate milk production of suckled cows, based on cow energy intake and milk yield during the nonsuckling period, or by first estimating calf milk consumption from their preweaning growth rate. These methods were also applied to conventionally managed, nonsuckled cows as validation step. With the first method, milk production of suckled cows was underestimated when using energy intake and milk yield during the nonsuckling period. With the second method, milk production of suckled cows estimated using calf growth aligned with the recorded machine milk yield of nonsuckled cows. The ~15 kg/day estimated calf milk consumption during the 13-week suckling period corresponded to the recorded milk yield difference between suckled and nonsuckled cows. These methods can be used for performance testing and sustainability analyses of cow-calf contact systems. estMP = estimated milk production; estMP adj. = estMP adjusted for the difference in actual and estimated milk consumption of NC calves.

In conventional dairy systems, cow and calf are commonly separated within 24 h after birth (Stěhulová et al., 2008), primarily to facilitate monitoring of calf colostrum and milk consumption, limiting disease transmission between cows and calves, and maximizing saleable milk yield (Flower and Weary, 2001; as reviewed by Beaver et al., 2019). In dairy cow-calf contact (**CCC**) systems, calves are reared with their dams or with foster cows. Variations of these systems exist in practice (Eriksson et al., 2022), often differing in whether full or only partial physical CCC is allowed and whether CCC spans the whole day or only part-time (Sirovnik et al., 2020). Saleable milk yield during the suckling period is lower in CCC systems than in conventional dairy systems due to calf consumption (Barth, 2020; van Zyl et al., 2025b). Milk intake of suckling calves cannot be measured but can be estimated by weighing calves before and after suckling (Fröberg et al., 2008). With full CCC, this technique is very labor-intensive and not feasible, as calves can suckle, defecate, urinate and consume solid feed throughout the day. Consequently, in whole-day systems with full CCC, the total milk production of the suckled cows is unknown and includes the milk yield delivered to the milking unit (or machine milk yield; **MMY**) and the milk consumed by the calf. Total milk production of suckled cows might be required for, for instance, performance testing of the cows (Bickelhaupt and Verwer, 2013; Kälber and Barth, 2014) and for determining economic and environmental effects of prolonged CCC (Knierim et al., 2020; Mogensen et al., 2022). In environmental studies, for example, GHG emission intensities are often expressed per unit of milk, but will be inflated when total milk production cannot be used. Therefore, this study aimed to estimate the total daily milk production of dairy cows with full CCC spanning the whole day using 2 different methods. First, individual milk production was estimated using the daily MMY during the suckling period and the ratio of daily energy intake to previous-day MMY calculated for the 8-wk postsuckling period, following the approach of Churakov et al. (2023). Second, individual milk production was estimated using the daily MMY during the suckling period and adding the calf milk consumption estimated based on their BW, growth, and energy intake from concentrates, following the approach of Quigley (2001a,b). These methods were also applied to cows and calves with no contact as a validation step.

Dairy cows of the Swedish Livestock Research Centre of the Swedish University of Agricultural Sciences in Uppsala were enrolled between October and November 2023, after approval by Uppsala Ethics Committee for Animal Research (diary number: 5.8.18-12179/2023). Inclusion criteria and detailed housing and management practices of the cows and calves have been reported by van Zyl et al. (2026) and van Zyl et al. (2025a), respectively. In short, the no contact (**NC**) group included 20 cows (5 primiparous cows of the Swedish Red breed [n = 3] and Swedish Holstein breed [n = 2], and 15 multiparous cows of the Swedish Red breed [n = 11] and Swedish Holstein breed [n = 4]) and their 22 calves (15 female including 2 twin pairs, 7 male) separated on average 13.5 h (12–17 h) after birth. The NC calves were dairy breed (n = 14) or dairy crossbred with beef breed (n = 8). The full contact (**FC**) group included 18 cows (6 primiparous cows of the Swedish Red breed [n = 4] and Swedish Holstein breed [n = 2], and 12 multiparous cows of the Swedish Red breed [n = 8] and Swedish Holstein breed [n = 4]) and their 19 calves (11 female, 8 male including 1 twin pair) that were allowed to have full CCC for minimum 10.6 ± 1.07 wk. The FC calves were dairy breed (n = 10) or dairy crossbred with beef breed (n = 9).

The NC cows were housed in a freestall barn after separation, whereas NC calves were group-housed elsewhere without further cow contact. The FC cow-calf pairs were moved to the same freestall barn as NC cows after ∼4 d together in individual calving boxes, and had access to a specific contact area where NC cows were not present. The FC calves had access to a calf creep, an area adjacent to the contact area where only the calves could enter. In the barn, cows followed a semi-controlled Feed First (DeLaval International AB, Tumba, Sweden) system: cows could freely access the feed alley, but were directed by a 3-way selection gate (Smart Selection Gate SSG, DeLaval, Tumba, Sweden) from the feed alley to either the automatic milking unit (VMS Classic, DeLaval, Tumba, Sweden), or NC cows to the lying cubicles and FC cows to the contact area. The FC cows could exit the contact area freely via a 1-way gate to the lying cubicles where NC cows were.

Cows had ad libitum access to a partial mixed ration provided in 20 individual roughage feeding bins with weighing scales (CRFI, BioControl A/S, Rakkestad, Norway). The partial mixed ration consisted of grass-clover silage, pelleted concentrates at 8 kg/cow per day and minerals at 75 g/cow per day. After the 19th wk of the experiment (cows at 15.2 ± 2.11 wk in lactation, on average), maize silage was also included in the ration, according to standard herd procedures. For the preweaning period, silage provided had a DM content of 39.8%, 150 g CP/kg DM, 403 g NDF/kg DM, 75 g ash/kg DM, and 11 MJ ME/kg DM. For the postseparation period, the contents were 39.2% DM, 129 g CP/kg DM, 388 g NDF/kg DM, 96 g ash/kg DM, and 11 MJ ME/kg DM. Cows received concentrates (EDEL Topp Single and EDEL Nova 190, AB Johan Hansson, Uppsala, Sweden) during milking and in the concentrate stations (FSC400, DeLaval, Tumba, Sweden). All diets were provided according to the Nordic feed evaluation system (NorFor; Volden, 2011). Individual roughage and concentrate intake was recorded continuously and registered in the management software (DelPro FarmManager 10.2, version 2023.5.4.9, DeLaval, Tumba, Sweden). Cow roughage intake data were cleaned before analyses by removing data where individual feeding rate was <0.02 kg/min or >2 kg/min or where feeding bin visits lasted >3 h (van Zyl et al., 2026), whereafter daily roughage and concentrate intake of all cows was calculated. The MMY per milking was registered in the management software, whereafter daily MMY was calculated for each cow. Milk samples of all cows were taken once every 4 wk during milking, whereafter the composition was analyzed (4.5% ± 0.76% fat for NC and 3.8% ± 0.80% for FC; 3.7% ± 0.33% protein in both treatments) and NC cow averages for the preweaning period were used for calculations in the current study. Milk lactose content was not analyzed and was based on a previous Swedish study (4.7%; Priyashantha et al., 2021).

During the preweaning period, NC calves received 8 L of whole milk daily (2 × 4 L; aligned with EFSA Panel on Animal Health and Animal Welfare et al., 2023) from an automatic milk taxi (Holm & Laue, Westerrönfeld, Germany) and had ad libitum access to grass-clover silage, concentrates (Idol, Lantmännen, Stockholm, Sweden), hay, and water. During the preweaning period, FC calves had ad libitum access to the lactating cow partial mixed ration in the calf creep, and to the same concentrates as NC calves, as well as hay and water. In both groups, calf concentrate was provided ad libitum in automatic concentrate stations (DeLaval Concentrate Station, Tumba, Sweden) recording individual concentrate supply, assumed to reflect actual intake (van Zyl et al., 2025a) and used to calculate daily concentrate intake. Calves were gradually weaned over 10 d, aiming for a minimum 12-wk suckling period: NC calves from 12.6 ± 1.22 wk of age via a gradual reduction in daily milk allowance, and FC calves from 13.0 ± 1.11 wk of age via fence-line contact with FC cows, allowing gradually reducing suckling opportunities (for more detail, see van Zyl et al., 2025a). The FC calves were permanently separated from the cows 1 wk after weaning, at 15.3 ± 1.16 wk of age.

Data available to estimate the daily milk production of FC cows (representing the removable milk, thus MMY and the milk consumed by the calf) were daily MMY, daily roughage and concentrate intake, and energy content of the roughage and concentrates for the cows. For the calves, available data were birth weight, BW at the start of weaning, daily concentrate intake, energy content of the concentrates, and (for NC calves) the amount of milk provided. Calf roughage intake data were not available and hence not included in the analyses.

In the first method for estimating daily milk production during the preweaning period, until the start of gradual weaning, recorded MMY and postseparation feed intake data of the cows (after FC cows were separated from their calves, wk 15 to 23) were used, together with feed intake from the preweaning period (Churakov et al., 2023). For this, the ratio of daily MMY to previous-day ME intake was calculated per cow per day. Subsequently, the median ratio per cow for wk 15 to 23 was calculated. Finally, for the preweaning period, the cow's ME intake of the previous day was multiplied by this calculated individual median ratio to estimate daily milk production (kg/d).

In the second method for estimating milk production during the preweaning period, calf BW, preweaning growth, and energy intake from concentrates were used, following the approach of Quigley (2001a,b), based on NRC (2001). Individual preweaning growth was calculated from birth weight and BW at the start of weaning. The following steps were taken to estimate daily individual milk consumption of all calves during the preweaning period:

1. ME required for maintenance (**ME_m_**; Mcal/d) = (0.086 × BW^0.75^)/0.825
2. ME required for growth (**ME_g_**; Mcal/d) = (0.84 × BW^0.355^ × ADG^1.2^ × 0.69)/0.652
3. ME content of the milk (**ME_milk_**; Mcal) = (0.092 × fat [%] + 0.057 × CP [%] + 0.0395 × lactose [%]).
4. ME intake from concentrates (Mcal/d) = ME content of the concentrates (3.035 Mcal/kg) × average individual intake (kg/d) during the suckling period
5. ME intake from milk (Mcal/d) = ME_m_ + ME_g_ − ME intake from concentrates
6. Individual milk consumption (kg/d) = ME intake from milk/ME_milk_
7. Adjust estimated FC calf milk consumption by the difference between the milk allowance of NC calves (8 L/d, thus 8.24 kg/d, based on a milk density of 1.03 kg/L) and their estimated milk consumption

The group-average estimated milk consumption of FC calves was subsequently added to the daily individual MMY of FC cows during the preweaning period to obtain the estimated daily milk production (kg/d). Using group-average estimated milk consumption of FC calves accounted for unbalanced dam-calf numbers and allosuckling.

Statistical analyses were performed in SAS (version 9.4, SAS Institute Inc., Cary, NC). Daily MMY and milk production estimated according to the first method of all cows during the preweaning (until the start of weaning) and postseparation period were analyzed with linear mixed models. In both models, fixed effects included treatment, period, cow breed, and parity class, and the interaction between treatment and period. Daily milk production of FC cows estimated according to the second method was analyzed during the preweaning period following a similar approach as described previously, but without period effects in the model. In all models, day was the repeated measure, cow the repeated subject, and the compound symmetry covariance structure was used. Univariate analysis of all model variables and residuals was performed to test for normality. Differences were significant when *P* < 0.05, and values are presented as LSM ± SEM. Daily MMY and daily milk production estimated via both methods were averaged per treatment and presented per week.

Average daily MMY of NC and FC cows and their estimated milk production based on ME intake are presented in Figure 1. Daily MMY and estimated milk production between wk 15 (after separation of FC cows and calves) and 23 did not differ between treatments (*P* ≥ 0.80), with milk production overestimated for both treatments. For NC cows, postseparation MMY was 36.11 ± 1.60 kg/d, estimated milk production was 37.00 ± 1.4 kg/d, and the average daily difference between estimated and recorded yield was 0.10 kg/d (95% CI: −0.39, 0.60). For FC cows, postseparation MMY was 36.19 ± 1.60 kg/d, estimated milk production was 37.45 ± 1.40 kg/d, and the average daily difference was 0.44 kg/d (95% CI: −0.05, 0.93). For the postseparation period, the estimation model based on the ME intake was thus close to true MMY, as Churakov et al. (2023) also concluded. For the preweaning period, MMY of NC cows was greater than MMY of FC cows (*P* < 0.001), and estimated milk production of NC and FC cows did not differ (*P* = 0.29). For NC cows, preweaning MMY was 37.73 ± 1.60 kg/d, estimated milk production was 33.33 ± 1.40 kg/d, and the average daily difference was −6.13 kg/d (95% CI: −6.67, −5.59). For FC cows, preweaning MMY was 24.74 ± 1.60 kg/d, estimated milk production was 35.30 ± 1.40 kg/d, and the average daily difference was 10.29 kg/d (9.70, 10.88). Multiparous cows had greater MMY (*P* < 0.001) and estimated milk production (*P* = 0.05) than primiparous cows. Suckled cows may have altered milk ejection to the milking unit, likely affecting their recorded MMY (Barth, 2020), which was not considered in the current study. The ∼1.97 kg/d higher estimated milk production of FC cows compared with NC cows is likely a result of their greater daily roughage intake during the preweaning period, as previously reported (van Zyl et al., 2026).

**Figure 1:**
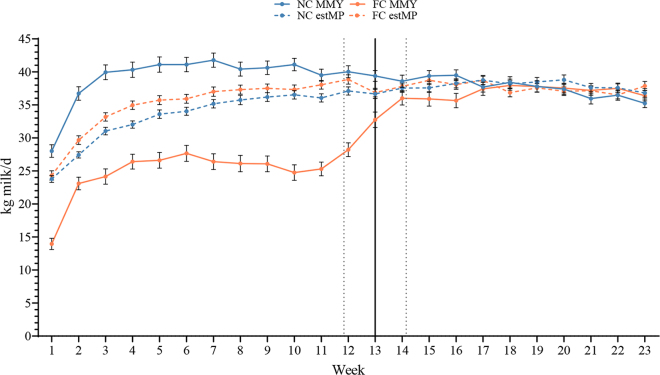
Machine milk yield (MMY) and estimated total milk production (estMP) of cows with no contact (NC) or full contact (FC) with their calves spanning the whole day. Milk production was estimated following the approach of Churakov et al. (2023). The average start of gradual weaning and its SD are represented by the vertical black line and the dotted lines, respectively. Values are presented as means ± SEM.

The underestimation of the milk production of NC cows during the preweaning period based on their ME intake was, however, neither expected nor found by Churakov et al. (2023). In both the current study and Churakov et al. (2023), the median ratio of daily MMY to previous-day ME intake calculated for the postseparation period was used to estimate milk production in the preweaning period. When using this median ratio of NC cows calculated during the preweaning period instead, the mean difference of their estimated milk production did not improve and was −5.98 kg/d (95% CI: −6.52, −5.44). This approach could not be applied to the FC cows due to the unrestricted suckling of the calves. A possible explanation for the underestimation is that the cows experienced a negative energy balance during the preweaning period. When in negative energy balance, energy intake fails to meet the energy required for maintenance and milk production (de Vries and Veerkamp, 2000). Cows compensate for this energy deficit by mobilizing body reserves. In a study conducted at the same research facilities as the current study (Andrée O'Hara et al., 2018), conventionally managed cows experienced a negative energy balance during the first 12 wk of lactation. It can be expected that cows in the current study also experienced a negative energy balance during the preweaning period, which was not accounted for in this method.

In the current study, the second method for estimating the daily milk production of the suckled FC cows was based on the preweaning growth of their calves (Figure 2). Milk consumption of FC calves was estimated at 14.91 ± 2.9 kg/d (or 14.48 L/d; mean ± SD), whereby the milk production of FC cows during the preweaning period was estimated at 37.94 ± 1.80 kg/d. Milk consumption of NC calves was estimated at 9.53 ± 2.4 kg/d, which is 1.29 kg/d more than what they were provided with (or 9.25 vs. 8.00 L/d). This discrepancy likely represents the energy obtained from the unrecorded roughage feed consumption, as ME intake from concentrates has already been deducted. Adjusting for the difference between estimated and actual NC calf milk consumption, milk consumption of FC calves was estimated at 13.62 ± 2.9 kg/d, whereby milk production of FC cows during the preweaning period was estimated at 36.65 ± 1.80 kg/d. Multiparous cows had greater estimated milk production (*P* < 0.001) than primiparous cows.

**Figure 2:**
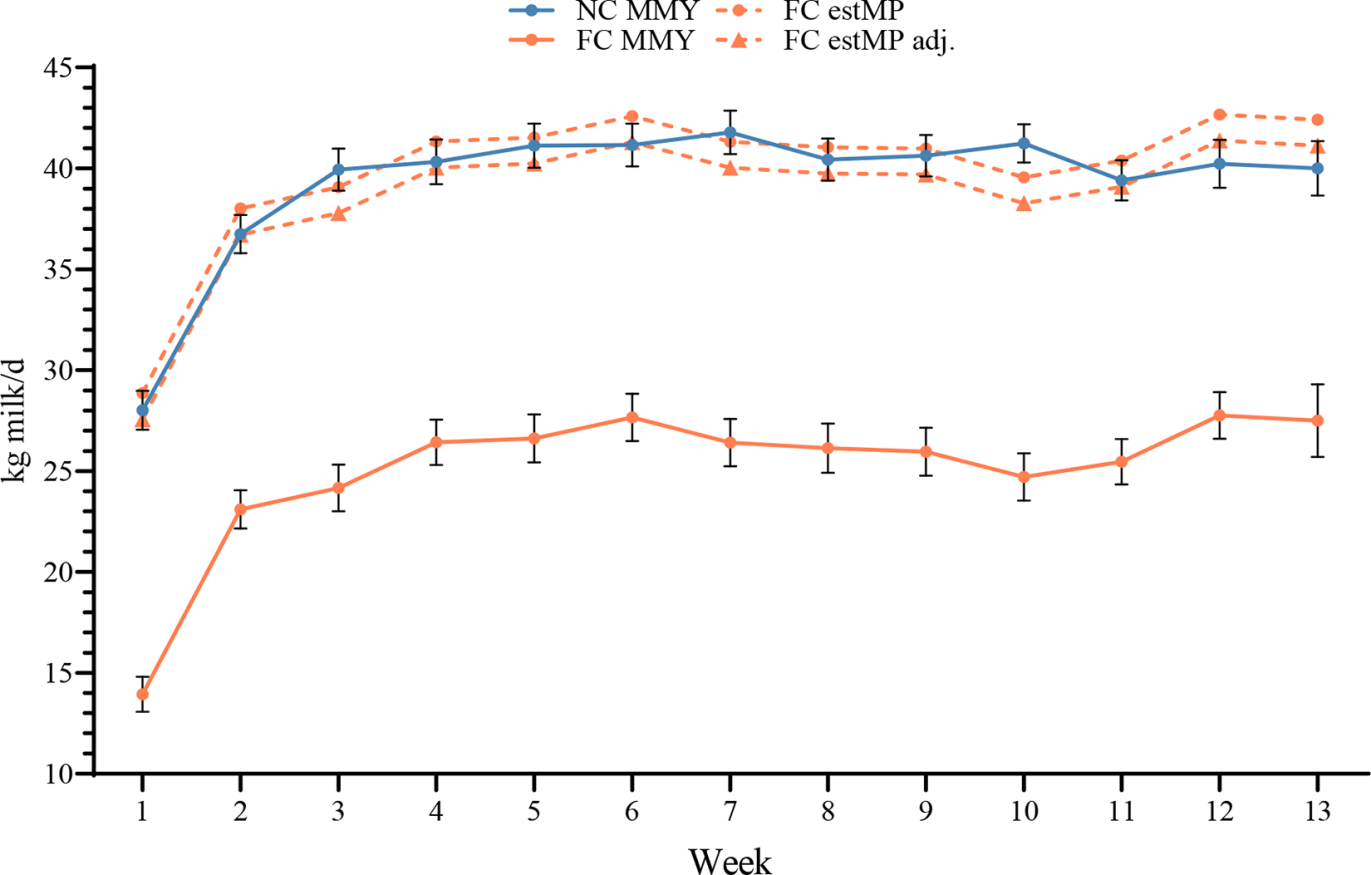
Machine milk yield (MMY) and estimated total milk production (estMP) of cows with no contact (NC) or full contact (FC) with their calves spanning the whole day. Milk production was estimated using the estimated calf milk consumption, following the approach of Quigley (2001a,b). The estMP adjusted for the difference in actual and estimated milk consumption of NC calves shown as estMP adj. Values are presented as means ± SEM.

Milk production of suckled FC cows estimated based on preweaning calf growth did not differ from the MMY of NC cows (*P* ≥ 0.59). Milk consumption of FC calves estimated from their preweaning growth likely represented their actual milk consumption well, as it closely corresponded to the difference in recorded MMY between treatments during the preweaning period. Additionally, it was comparable to the 16 L/d milk consumption of suckler calves (Scholz et al., 2001), and the higher estimated consumption was reflected by a greater preweaning growth in FC than NC calves (1.4 vs. 1.0 kg/d), as in our previous study on the same calves (van Zyl et al., 2025a). Although the energy intake from roughage could not be included in estimations, FC calves were expected to have a delayed start in roughage intake compared with NC calves, as they could not observe the cows feeding, and therefore there was no imitation effect (Costa et al., 2014). Moreover, concentrate intake of FC calves increased after the start of weaning only, later than NC calves (van Zyl et al., 2025a). The average estimated milk consumption was used, as opposed to individual estimations, because of allosuckling in the FC group (personal observations). Without allosuckling occurring, individual milk consumption estimates can be used to estimate individual milk production, providing more insight into the total milk production of individual cows and further application in specialized breeding strategies.

Future research can apply the methods evaluated in economic and environmental sustainability analyses of CCC systems, for example to quantify total milk output and the share consumed by calves versus sold, and to calculate GHG emission intensities. The approach is also relevant for research on CCC systems under different conditions (e.g., type of CCC, calf breed, preweaning growth, feeding regimens). More extensive data recording would improve estimates of calf milk consumption and cow milk production. For instance, recording calf roughage intake during the preweaning period would improve intake estimates, and more frequent BW measurements of suckling calves would allow milk consumption to be estimated at multiple points, reflecting calves' increasing milk consumption over time. In the current study, FC calves likely consumed less milk than the estimated ∼15 kg/d early on and more in the last suckling weeks. Additionally, because calves may be able to evacuate milk fat more efficiently than the milking unit (S⊘rby et al., 2024), the milk ingested by FC calves throughout the day could have differed from FC milk sampled during machine milking. Therefore, NC milk composition was used in the calculations. Using the actual composition of ingested milk might improve estimation accuracy. Finally, limited data availability on commercial farms, such as missing calf concentrate intake records or infrequent BW measures, may restrict the practical application of this method.

In conclusion, both methods evaluated could estimate milk production of suckled FC cows. In the first method, milk production was underestimated when using ME intake and milk yield during the nonsuckling period, possibly due to not accounting for body reserve mobilization. In the second method, milk production of suckled cows estimated using calf growth aligned with the recorded MMY of nonsuckled cows. Milk consumption of CCC calves was estimated at ∼15 kg/d during the first 13 wk of life, corresponding to the recorded milk yield difference between CCC and nonsuckled cows.

## References

[bib1] Andrée O’Hara E., Omazic A., Olsson I., Båge R., Emanuelson U., Holtenius K. (2018). Effects of dry period length on milk production and energy balance in two cow breeds. Animal.

[bib2] Barth K. (2020). Effects of suckling on milk yield and milk composition of dairy cows in cow–calf contact systems. J. Dairy Res..

[bib3] Beaver A., Meagher R.K., von Keyserlingk M.A.G., Weary D.M. (2019). Invited review: A systematic review of the effects of early separation on dairy cow and calf health. J. Dairy Sci..

[bib4] Bickelhaupt C., Verwer C. (2013). Investigating Marketing Opportunities for Dairy Products from Dam Rearing Systems Summary of the similarly titled report. Louis Bolk Institute. Accessed Nov. 18, 2025. https://www.louisbolk.nl/sites/default/files/publication/pdf/2818.pdf.

[bib5] Churakov M., Eriksson H.K., Agenäs S., Ferneborg S. (2023). Proposed methods for estimating loss of saleable milk in a cow-calf contact system with automatic milking. J. Dairy Sci..

[bib6] Costa J.H.C., Daros R.R., von Keyserlingk M.A.G., Weary D.M. (2014). Complex social housing reduces food neophobia in dairy calves. J. Dairy Sci..

[bib7] de Vries M.J., Veerkamp R.F. (2000). Energy balance of dairy cattle in relation to milk production variables and fertility. J. Dairy Sci..

[bib8] Eriksson H., Fall N., Ivemeyer S., Knierim U., Simantke C., Fuerst-Waltl B., Winckler C., Weissensteiner R., Pomiès D., Martin B., Michaud A., Priolo A., Caccamo M., Sakowski T., Stachelek M., Spengler Neff A., Bieber A., Schneider C., Alvåsen K. (2022). Strategies for keeping dairy cows and calves together—A cross-sectional survey study. Animal.

[bib9] Flower F.C., Weary D.M. (2001). Effects of early separation on the dairy cow and calf: 2. Separation at 1 day and 2 weeks after birth. Appl. Anim. Behav. Sci..

[bib10] Fröberg S., Gratte E., Svennersten-Sjaunja K., Olsson I., Berg C., Orihuela A., Galina C.S., García B., Lidfors L. (2008). Effect of suckling ('restricted suckling’) on dairy cows’ udder health and milk let-down and their calves’ weight gain, feed intake and behaviour. Appl. Anim. Behav. Sci..

[bib11] Kälber T., Barth K. (2014). Practical implications of suckling systems for dairy calves in organic production systems—A review. Landbauforschung (Braunschw).

[bib12] Knierim U., Wicklow D., Ivemeyer S., Möller D. (2020). A framework for the socio-economic evaluation of rearing systems of dairy calves with or without cow contact. J. Dairy Res..

[bib13] Mogensen L., Kudahl A., Kristensen T., Bokkers E.A.M., Webb L.E., Vaarst M., Lehmann J. (2022). Environmental impact of dam-calf contact in organic dairy systems: A scenario study. Livest. Sci..

[bib14] EFSA Panel on Animal Health and Animal Welfare, Nielsen, S.S., Alvarez J., Bicout D.J., Calistri P., Canali E., Drewe J.A., Garin-Bastuji B., Gonzales Rojas J.L., Gortázar Schmidt C., Herskin M., Michel V., Miranda Chueca M.Á., Padalino B., Roberts H.C., Spoolder H., Stahl K., Velarde A., Viltrop A., De Boyer des Roches A., Jensen M.B., Mee J., Green M., Thulke H.-H., Bailly-Caumette E., Candiani D., Lima E., Mosbach-Schulz O., Van der Stede Y., Vitali M., Winckler C. (2023). Welfare of calves. EFSA J. 21:e07896. https://doi.org/10.2903/j.efsa.2023.7896.

[bib15] NRC (National Research Council) (2001). Nutrient Requirements of Dairy Cattle. 7the rev. ed. National Academies Press, Washington, DC. Accessed Mar. 15, 2026. https://guelphdhmcp.ca/wp-content/uploads/2018/03/NRC-2001.pdf.

[bib16] Priyashantha H., Lundh Å., Höjer A., Bernes G., Nilsson D., Hetta M., Saedén K.H., Gustafsson A.H., Johansson M. (2021). Composition and properties of bovine milk: A study from dairy farms in northern Sweden; Part II. Effect of monthly variation. J. Dairy Sci..

[bib17] Quigley J.D. (2001). Calf Note #71-NRC Energy Requirements for Calves Fed Milk or Milk Replacer. Accessed Oct. 22, 2025. https://calfnotes.com/en/2001/05/09/calf-note-071-nrc-energy-requirements-for-calves-fed-milk-or-milk-replacer/.

[bib18] Quigley, J.D. (2001). Calf Note #72-NRC Energy Requirements for Calves Fed Milk or Milk Replacer Plus Starter. Accessed Oct. 22, 2025. https://calfnotes.com/en/2001/05/27/calf-note-072-nrc-energy-requirements-for-calves-fed-milk-or-milk-replacer-plus-starter/.

[bib19] Scholz H., Kovács A.Z., Stefler J., Fahr R.-D., von Lengerken G. (2001). Milchleistung und -qualität von Fleischrindkühen während der Säugeperiode. Arch. Tierzucht.

[bib20] Sirovnik J., Barth K., de Oliveira D., Ferneborg S., Haskell M.J., Hillmann E., Jensen M.B., Mejdell C.M., Napolitano F., Vaarst M., Verwer C.M., Waiblinger S., Zipp K.A., Johnsen J.F. (2020). Methodological terminology and definitions for research and discussion of cow-calf contact systems. J. Dairy Res..

[bib21] Sørby J., Johnsen J.F., Kischel S.G., Ferneborg S. (2024). Effects of two gradual debonding strategies on machine milk yield, flow and composition in a cow-driven cow-calf contact system. J. Dairy Sci..

[bib22] Stěhulová I., Lidfors L., Špinka M. (2008). Response of dairy cows and calves to early separation: Effect of calf age and visual and auditory contact after separation. Appl. Anim. Behav. Sci..

[bib23] van Zyl C.L., Bokkers E.A.M., Aarts Y.J.M., Bus J.D., Kemp B., Eriksson H., van Knegsel A.T.M. (2026). Roughage feeding patterns of dairy cows in a cow-calf contact system with automatic milking. Appl. Anim. Behav. Sci..

[bib24] van Zyl C.L., Bokkers E.A.M., Kemp B., Agenäs S., van Knegsel A.T.M. (2025). Growth and metabolism of calves in a dairy cow-calf contact system with gradual weaning and separation. J. Dairy Sci..

[bib25] van Zyl C.L., Eriksson H.K., Bokkers E.A.M., Kemp B., van Knegsel A.T.M., Agenäs S. (2025). Consequences of weaning and separation for feed intake and milking characteristics of dairy cows in a cow-calf contact system. J. Dairy Sci..

[bib26] Volden H. (2011). *NorFor*—The Nordic feed evaluation system. Wageningen Academic Publishers..

